# LPS and IL-8 activated umbilical cord blood-derived neutrophils inhibit the progression of ovarian cancer

**DOI:** 10.7150/jca.41035

**Published:** 2020-05-18

**Authors:** Qi Liu, Weihong Yang, Ning Luo, Jie Liu, Yuliang Wu, Jinye Ding, Caixia Li, Zhongping Cheng

**Affiliations:** 1 Department of Obstetrics and Gynecology, Shanghai Tenth People's Hospital, Tongji University School of Medicine, Shanghai 200072, China; 2 Institute of Gynecological Minimally Invasive Medicine, Tongji University School of Medicine, Shanghai, China

**Keywords:** Immunotherapy, Ovarian cancer, Neutrophils, Umbilical cord blood (UCB)

## Abstract

**Background**: Immunotherapy including immune checkpoint blockade, cancer vaccines, and adoptive cell therapy. However, no immune therapies support ovarian cancer. It is not clear whether the neutrophils, the component of the immune system derived from umbilical cord blood play a role in inhibiting the progression of ovarian cancer.

**Methods**: We investigate the impact of LPS and IL-8 activated neutrophils derived from umbilical cord blood(UCB)on ovarian cancer progression. After co-culture LPS and IL-8 activated UCB-derived neutrophils with ovarian cancer cell line SKOV3 and OVCAR3, CCK8, Transwell assay, and Flow Cytometry was performed to detect cell proliferation, migration, invasion, and apoptosis of ovarian cancer cell lines SKOV3 and OVCAR3. Furthermore, RT-PCR and western blotting assay were used to analyze the mechanism of metastasis and apoptosis of ovarian cancer cell lines respectively to support previous function experiments.

**Results**: We demonstrate LPS and IL-8 activated neutrophils derived from umbilical cord blood inhibit proliferation, invasion migration and promote apoptosis of SKOV3 and OVCAR3. Meanwhile, LPS and IL-8 activated UCB-derived neutrophils significantly decreased BAX and increased BCL2 expression in SKOV3 and OVCAR3 which account for the mechanism of apoptosis. Moreover, LPS and IL-8 activated UCB derived neutrophils significantly up-regulated E-cadherin and downregulated N-cadherin, MMP2 expression in SKOV3 and OVCAR3.

**Conclusion**: Taken together, these results approved that LPS and IL-8 activated neutrophils from UCB may be the novel strategy in immune therapy for ovarian cancer.

## Background

Ovarian cancer is one of the most lethal gynecologic malignancies due to later stages of diagnosis and reduced therapeutic efficacy 1. The standard management of ovarian cancer combines cytoreductive surgery and chemotherapy. Although rapid advances exist in multiple therapy strategies, the clinical outcome has not been improved in ovarian cancer patients, so novel therapeutic strategies are required to archive successful management of ovarian cancer. Consequently, new treatment strategies such as immunotherapy are appearing. Numerous investigations on immune checkpoint blockade, cancer vaccines, adoptive cell therapy immune therapies for ovarian cancer 2, 3.

In this context, neutrophils as a substantial proportion of the innate immune system have been reported exerts anti-tumorigenic roles in the tumor microenvironment 4. Tumor-associated neutrophils have variously been shown to robustly produce a variety of toxic compounds and can induce tumor cell cytolysis or cytostasis in vitro, which suggests that in certain circumstances they might oppose tumorigenesis 5. The mechanisms of neutrophils killing cancer cells may be direct cytotoxicity toward tumor cells 6. Neutrophils can be polarized into different phenotypes according to various cytokines secreted by cancer cells in tumor micro environment 7, 8.

In this regard, adoptive cell therapy (ACT) exploiting allogeneic neutrophils was first established in our laboratory to detect a novel approach against ovarian cancer. However, activated and sufficient neutrophils numbers are needed for effective neutrophils-based adoptive cell therapy. Generally, peripheral blood is a rich resource of allogeneic neutrophils. But, peripheral blood is precious and yields low neutrophils numbers, cannot satisfy higher neutrophils numbers needed for adoptive transfer. Alternatively, haematopoietic stem and progenitor cells (HSPC) 9 or induced pluripotent stem cells (iPSC) 10 can be used for generate neutrophils. However, these techniques consume cost plentiful manpower and material resources. So, easily available and abundant homogeneous umbilical cord blood-derived neutrophils products are needed for immunotherapy.

In this study, we examine the impact of LPS and IL-8 activated umbilical cord blood-derived neutrophils on ovarian cancer cells. We demonstrate whether the LPS and IL-8 activated neutrophils derived from umbilical cord blood could inhibit the progression of ovarian cancer.

## Methods

### Isolation neutrophils from UCB and activation

Umbilical cord blood (UCB) units were collected in CB-collect bags (Fresenius Kabi) at cesarean sections after a full-term pregnancy and informed consent was obtained from the mother (CMO 2014-226). Neutrophils were isolated from UCB after 1077 and 1119 density-gradient centrifugation (Sigma). After isolation, neutrophils were directly used for the next experiments. Then neutrophils were cultured in 24 well plates within 1640 medium supplemented with 10% fetal serum. And fresh isolated CD11b+ neutrophils were stimulated with LPS (100ng/ml) (peprotech) and IL-8 (10ng/ml) (protect) for 24h respectively as the experimental groups, at the same time PBS as the control group.

### Cell lines and co-culture model

Human ovarian cancer cell lines (SKOV3, OVCAR3) were maintained in RPMI 1640 medium supplemented with 10% fetal bovine serum and 1% penicillin-streptomycin. Cells were cultured in a 5% CO2 incubator at 37℃. The mediums were replaced routinely every 2-3 days. After neutrophils were stimulated by LPS or IL-8 for 24h, neutrophils were transferred into 6- well cell culture plates seeded with SKOV3 or OVCAR3 cells in advance. After co-cultured for 16 h, the ovarian cancer cells were collected for follow-up assays.

### Cell proliferation assay

After co-culture, SKOV3, and OVCAR3 were digested and seeded in 96-well plates.

Viability of ovarian cancer cell lines was detected by cell counting kit 8 according to manufacturer's instruction at 0h, 12 h, 24h, 48 h, and 72 h, respectively. Independent experiments were repeated at least three times.

### Migration and Invasion assay

Migration and invasion of SKOV3 and OVCAR3 cells were assessed by transwell assay.5*10^4^ SKOV3 and OVCAR3 cells were seeded into the upper chamber of Matrigel-coated polycarbonate membrane filters. Then, UCB-derived neutrophils added to the lower chamber as a chemoattractant. Cells that had migrated to the lower chambers at 24 h were fixed with 4% paraformaldehyde for 30 min and stained with 0.1% crystal violet for 30 min. Three low-magnification areas were randomly selected, and the number of migrated cells was counted.

### Flow cytometry

PE-CD11b (BD, USA) antibody was used for flow cytometry. Isolated cells in UCB were stained with PE-conjugated monoclonal anti-human CD11b to identify the purity of neutrophils. After stained 30min, samples were analyzed by flow cytometry (FACS Calibur, BD Biosciences, San Jose, CA, USA) FlowJo software was used to analyze data. Annexin-V-FITC apoptosis detection kit (BD, Franklin Lakes, NJ, USA) was used to perform cell apoptosis assay.

### Quantitative RT-PCR

Total RNA was extracted from the ovarian cancer cell line after co-culture with UCB-derived neutrophils with TRIzol Reagent (Invitrogen) according to the manufacture's protocol. Reverse Transcription Kit was used to synthesizing cDNA. RT-PCR was done using the SYBR Green agent. GAPDH was used as control.

### Western blot

Sodium dodecyl sulfate-polyacrylamide was used to separate protein, then transferred to PVDF membranes. The membranes were incubated with antibodies against E-cadherin, N-cadherin, MMP2, BAX, BCL2 respectively. GAPDH antibody was used as a control. Then, horseradish peroxidase-conjugated secondary antibodies were added to membranes. ECL Western Blotting Detection System was used to detect proteins and the band was measured with LabWorks.

### Statistical analysis

Cell experiments were performed in triplicate. GraphPad Prism Version 5.0 and SPSS 19.0 were performed to analyze data that were presented as value ± standard deviation. Comparisons between 2 groups were performed by the standard Student's t-test. p<0.05 was considered to indicate a significant difference (*p< 0.05, **p≤ 0.01, ***p≤ 0.001).

## Results

### *Ex vivo* generation of high purity UCB-derived neutrophils

**Fig 1:**
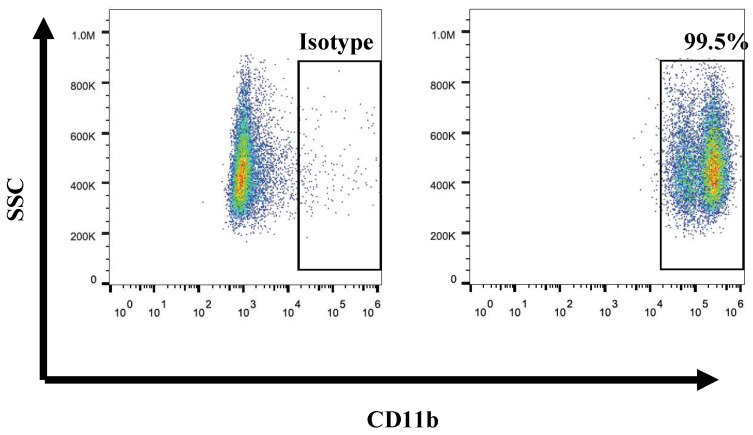
Flow cytometry showed the purity of UCB-derived neutrophils is over 90%.

Previous studies have reported numerous methods of isolated neutrophils from peripheral blood or tissues. In this study, we first isolated neutrophils from umbilical cord blood by concentration method. And we determined the purity of isolation neutrophils from umbilical cord blood by using the CD11b marker. Results revealed that the purity of the neutrophils was up to 95% (Fig. 1). It is indicated that we can isolate high purity of neutrophils from umbilical cord blood. On average, 1*10^7^ CD11b+ neutrophils can be isolated from every 5ml umbilical cord blood. Furthermore, as the expansion of neutrophils was unavailable, we take fresh umbilical cord blood every time religiously.

### LPS and IL-8 activated UCB-derived neutrophils inhibit proliferation of ovarian cancer cell lines

**Fig 2:**
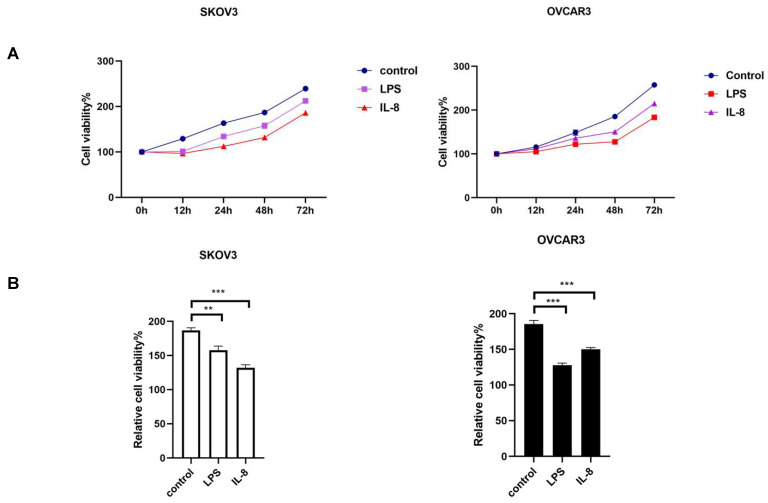
(A)The picture depicts the effect of LPS and IL-8 stimulated UCB-derived neutrophils on cell viability of SKOV3 and OVCAR3 as compared to control at 0h, 12h, 24h, 48h, 72h by CCK-8 assay. (B)Histograms showed the effect of stimulated UCB-derived neutrophils on cell viability of SKOV3 cells and OVCAR3 cells at 48h. *p < 0.05, **p < 0.01, ***p < 0.001.

After co-culture LPS and IL-8 stimulated UCB-derived neutrophils with ovarian cancer cell line SKOV3 and OVCAR3 for 16h respectively, medium including neutrophils was discarded, then CCK8 was used to assess the proliferation of SKOV3 and OVCAR3 at 0h, 12h, 24h, 48h, 72h respectively. Results revealed that the LPS stimulated group inhibited cellular proliferation at both SKOV3 and OVCAR3 compared with control (Fig. 2A, B). Similarly, IL-8 stimulated neutrophils inhibited proliferation of both SKOV3 and OVCAR3 compared with control (Fig. 2A, B). The results indicated that LPS and IL-8 stimulated UCB-derived neutrophils to suppress ovarian cancer cell proliferation.

### LPS and IL-8 activated UCB-derived neutrophils reduces the motility of ovarian cancer cell lines

**Fig 3:**
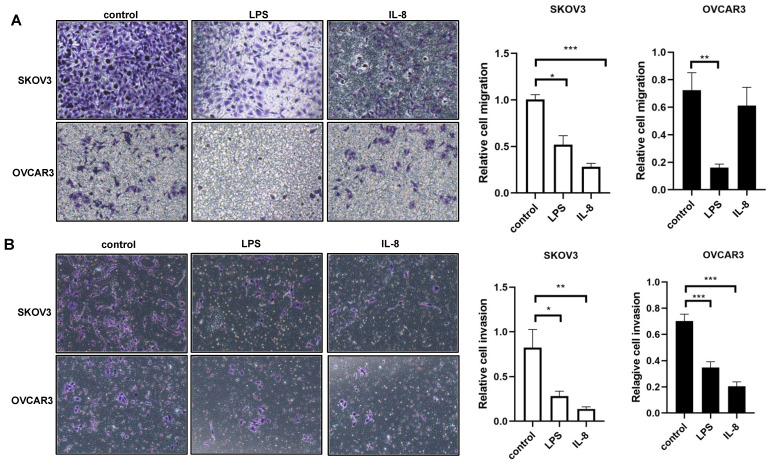
LPS and IL-8 stimulated UCB-derived neutrophils alters motility of ovarian cancer cells. (A)Representative photomicrographs show the reduction in SKOV3 and OVCAR3 migration through transwell membrane after co-culture with stimulated UCB-derived neutrophils compared to control. Histogram depicts the relative migration of treated SKOV3 and OVCAR3 cells compared with control. (B)Representative photomicrographs show the reduction in SKOV3 and OVCAR3 invasion through transwell membrane after co-culture with stimulated UCB-derived neutrophils compared to control. Histogram depicts the relative invasion of treated SKOV3 and OVCAR3 cells compared with control. *p < 0.05, **p < 0.01, ***p < 0.001

We next ask whether LPS and IL-8 stimulated UCB-derived neutrophils could induce motility of ovarian cancer cell line SKOV3 and OVCAR3.To answer this question, migration and invasion assay were performed to further assess the motility change of SKOV3 and OVCAR3. LPS stimulated UCB-derived neutrophils resulted in a reduction in migration and invasion ability of SKOV3 and OVCAR3 respectively compared with control (Fig. 3A, B). Similarly, IL-8 stimulated UCB-derived neutrophils resulted in a reduction in migration and invasive ability of SKOV3 and OVCAR3 respectively compared with control (Fig. 3A, B). These results demonstrated that LPS and IL-8 activated UCB-derived neutrophils could inhibit the motility of ovarian cancer cells.

### LPS and IL-8 activated UCB-derived neutrophils induce apoptosis in ovarian cancer cell lines

**Fig 4:**
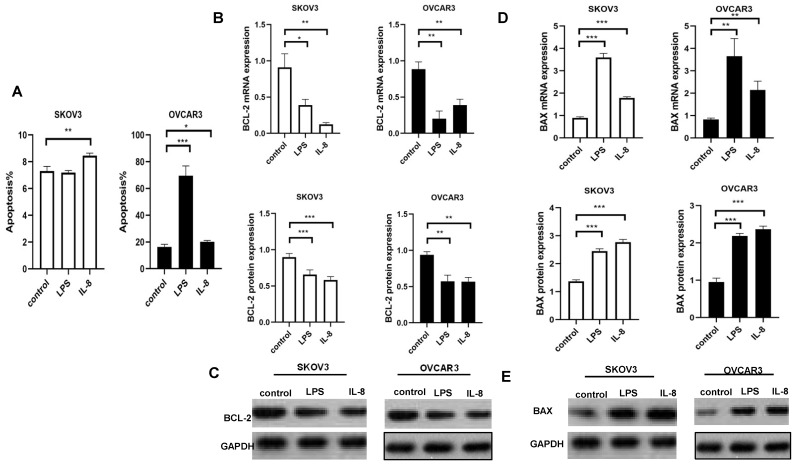
LPS and IL-8 stimulated UCB-derived neutrophils induced apoptosis of ovarian cancer cells. (A) Histogram depicts the apoptosis percentage of SKOV3 and OVCAR3 after co-culture with stimulated UCB-derived neutrophils. (B) Effect of LPS and IL-8 stimulated UCB-derived neutrophils on BCL-2 mRNA and protein expression in SKOV3 and OVCAR3 cells. (C)Representative photomicrographs show the expression of BCL-2 in SKOV3 and OVCAR3 by western blot. (D) Effect of LPS and IL-8 stimulated UCB-derived neutrophils on BAX mRNA and protein expression in SKOV3 and OVCAR3 cells. (E)Representative photomicrographs show the expression of BAX in SKOV3 and OVCAR3 by western blot.*p < 0.05, **p < 0.01, ***p < 0.001

We used flow cytometry to indicate the effect of LPS and IL-8 activated UCB-derived neutrophils on apoptosis of both SKOV3 and OVCAR3. We discarded co-culture medium including neutrophils, then flow cytometry assay was performed to further assess apoptosis of SKOV3 and OVCAR3.LPS stimulated UCB-derived neutrophils induced apoptosis of OVCAR3, but no changes were observed in SKOV3 (Fig. 4A). IL-8 stimulated UCB-derived neutrophils apoptosis of SKOV3 and OVCAR3 (Fig. 4A). Interestingly, LPS-stimulated UCB-derived neutrophils induced significantly higher apoptosis in OVCAR3 than SKOV3. It may be explained by the heterogeneous cancer cell lines.

### LPS and IL-8 activated UCB-derived neutrophils affect apoptosis-related signaling expression in ovarian cancer cell lines

To better investigate the effects of LPS and IL-8 activated UCB-derived neutrophils on ovarian cancer cells, we abstract mRNA and protein of SKOV3 and OVCAR3 respectively. Then, RT-PCR and Western Blot were used to analyze levels of apoptosis signaling expression. Subsequently,anti-apoptotic marker BAX and pro-apoptotic markers BCL-2were detected in both SKOV3 and OVCAR3. Results revealed that LPS stimulated UCB-derived neutrophils up-regulated pro-apoptotic markers BAX as well as down-regulated anti-apoptotic marker BCL2 in both mRNA and protein levels in SKOV3 and OVCAR3 compared with control(Fig.4B, C, D, E). Similarly, IL-8 stimulated UCB-derived neutrophils up-regulated pro-apoptotic markers BAX as well as down-regulated anti-apoptotic marker BCL2 in both mRNA and protein levels in SKOV3 and OVCAR3 compared with control(Fig.4B, C, D, E). It indicated that LPS and IL-8 activated UCB-derived neutrophils exerted anti-tumor effects on ovarian cancer cell lines.

### LPS and IL-8 activated UCB-derived neutrophils altered the expression of metastasis related signaling genes (proteins) in ovarian cancer cell lines

**Fig 5:**
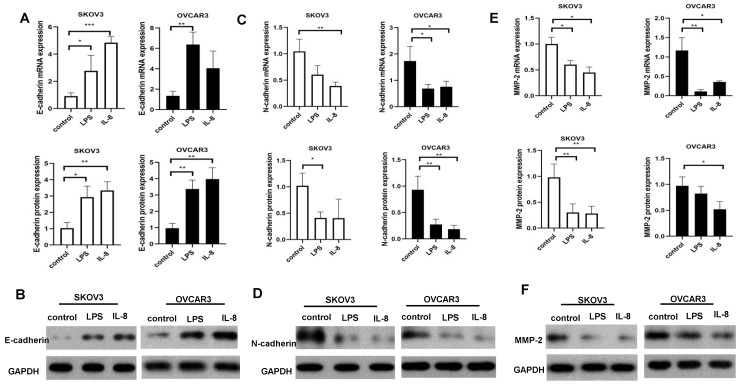
Effect of LPS and IL-8 stimulated UCB-derived neutrophils on E-cadherin, N-cadherin, MMP-2 mRNA and protein expression in SKOV3 and OVCAR3 cells. (A)RT-PCR and western blot performed to the analysis of the E-cadherin genes and protein level in SKOV3 and OVCAR3.GAPDH as a loading control. (B) Representative photomicrographs show the expression of E-cadherin in SKOV3 and OVCAR3 by western blot. (C)RT-PCR and western blot performed to the analysis of the N-cadherin genes and protein level in SKOV3 and OVCAR3. GAPDH as a loading control. (D) Representative photomicrographs show the expression of N-cadherin in SKOV3 and OVCAR3 by western blot. (E)RT-PCR and western blot performed to the analysis of the MMP-2 genes and protein level in SKOV3 and OVCAR3. GAPDH as a loading control. (F)Representative photomicrographs show the expression of N-cadherin in SKOV3 and OVCAR3 by western blot.*p < 0.05, **p < 0.01, ***p < 0.001

To further determine the mechanism of LPS and IL-8 activated UCB-derived neutrophils regulate ovarian cancer cell invasion and migration, RT-PCR and western blot were performed to detect metastasis related signaling expression E-cadherin, N-cadherin, MMP-2 in SKOV3 and OVCAR3. Our data showed that LPS stimulated UCB-derived neutrophils up-regulated E-cadherin mRNA and protein in SKOV3 and OVCAR3 (Fig. 5A, B). Moreover, LPS stimulated UCB-derived neutrophils down-regulated N-cadherin protein level in SKOV3, and both N-cadherin mRNA and protein in OVCAR3 (Fig. 5C, D). LPS stimulated UCB-derived neutrophils down-regulated MMP-2 both mRNA and protein in SKOV3 and down-regulated protein level of OVCAR3 (Fig. 5E, F). Furthermore, IL-8 stimulated UCB-derived neutrophils up-regulated E-cadherin mRNA and protein expression in SKOV3, and up-regulated E-cadherin protein level in OVCAR3 (Fig. 5A, B). IL-8 stimulated UCB-derived neutrophils down-regulated N-cadherin protein level in of SKOV3 and downregulated N-cadherin mRNA and protein expression in OVCAR3 (Fig. 5C, D). IL-8 stimulated UCB-derived neutrophils down-regulated MMP-2 both mRNA and protein in SKOV3 and OVCAR3 (Fig. 5E, F). It indicated that LPS and IL-8 stimulated UCB-derived neutrophils to promote mesenchymal-epithelial transition of ovarian cancer cells to inhibit migration and invasion.

## Discussion

Neutrophils involving the tumor-bearing model have the antitumor and antimetastatic potential 11, 12. Neutrophils infiltrating cancer tissues have antitumor phenotype 13. In this study, we evaluate the possibility of neutrophils based adoptive cell therapy (ACT) exploiting allogeneic immune cells for immunotherapy. As the clinical potential of umbilical cord blood-derived stem and progenitor cells focus on various animal and human transplantation studies such as therapeutic utility of cultured hematopoietic stem cells in large animals and humans 14, we first use neutrophils derived from umbilical cord blood, which present numerous advantages as abundant source of neutrophils.

As neutrophils can be polarized into N1 or N2 phenotype which was associated with enhanced release of neutrophil chemotactic factors from tumor cells 15, 16, we added inflammatory mediators LPS and cytokines IL-8 into UCB-derived neutrophils to generate activated neutrophils. LPS can trigger the dominant TLR4 and its activation by LPS lead to the activation of neutrophils 17. IL-8 activated neutrophils releasing specific proteases and a heparanase that hydrolyze components of the extracellular matrix (ECM) 18. We found that LPS and IL-8 activated UCB-derived neutrophils exhibit anti-tumor specialty. LPS and IL-8 may stimulate neutrophils turning into the N1 phenotype. The mechanisms of LPS and IL-8 activated neutrophils anti-tumor and the phenotype of activated neutrophils in this study need further study.

Proliferation, invasion, migration and cell death reflected the biology of the cancer cells. Notably, we observed LPS and IL-8 activated UCB-derived neutrophils could inhibit the bioactivity of ovarian cancer. LPS and IL-8 activated UCB-derived neutrophils inhibited proliferation, migration, and invasion of SKOV3 and OVCAR3. Furthermore, our study dissects that UCB-derived neutrophils suppressed ovarian cancer cells metastasis through regulation E-cadherin, N-cadherin, and MMP-2 which were mesenchymal-epithelial transition-related signaling. E-cadherin, N-cadherin, MMP-2 are key molecules to be involved in the mesenchymal-epithelial transition and cell invasion 19, 20. Our study showed that LPS and IL-8 stimulated UCB-derived neutrophils increased E-cadherin expression and decreased N-cadherin, MMP-2 expression in ovarian cancer cells. Thus, LPS and IL-8 stimulated UCB-derived neutrophils to inhibit invasion and migration by down-regulating N-cadherin, MMP-2 and up-regulating E-cadherin expression in ovarian cancer cells.

We found that umbilical cord blood-derived neutrophils activated by LPS and IL-8 induce apoptosis of SKOV3 and OVCAR3. However, LPS and IL-8 stimulated UCB-derived neutrophils resulted in different percentages of apoptosis of SKOV3 and OVCAR3. The different SKOV3 and OVCAR3 responses to LPS or IL-8 stimulated neutrophils can be explained by the genetic diversity of cancer cell lines. Genetic ancestry can impact the response to therapy, and cancer disparities result in biological and molecular aspects variation, even receipt of treatment 21-23. Germline variants exist in several commonly used cancer cell lines by examined ancestry 24, 25. Molecular classification, the variable of genetic ancestry should be considered according to the design and results of experiments. It is indicated that genetic background impact therapy response.

We further detect the mechanism of ovarian cancer apoptosis induction by UCB-derived neutrophils. Pro-apoptotic BAX and anti-apoptotic BCL2 are critical factors for initiating apoptosis via the mitochondria 26, 27. Mitochondria-mediated cell apoptosis is the major apoptotic pathway and is most commonly mediated by a variety of protein-membrane and protein-protein interactions of the B-cell lymphoma 2 protein (BCL2) family. BCL2-associated X (BAX), a member of the BCL2 family, is a pro-apoptotic protein. Results showed that LPS and IL-8 activated UCB-derived neutrophils decreased BCL2 expression and increased BAX expression of SKOV3 and OVCAR3. It indicated that LPS and IL-8 activated UCB-derived neutrophils may trigger the mitochondrial apoptotic pathway in ovarian cancer cells. However, the BCL2 family proteins are regulated by many signaling pathways. In our study, UCB-derived neutrophils changed the expression of BCL2 family proteins; however, there was no evidence showing that UCB-derived neutrophils could directly regulate their expression levels. We concluded that LPS and IL-8 activated UCB-derived neutrophils regulated the apoptosis proteins of ovarian cancer cells indirectly through other signaling pathways.

For the first time, we present the relationship between LPS and IL-8 activated umbilical cord blood-derived neutrophils and ovarian cancer. In conclusion, our results give the primary detection that LPS and IL-8 activated neutrophils from umbilical cord blood could inhibit the progression of ovarian cancer cells. Our findings provide strong evidence of UCB-derived neutrophils based immunotherapy against ovarian cancer.

## Conclusions

In summary, this investigation demonstrated that LPS and IL-8 activated UCB-derived neutrophils can inhibit proliferation, migration, and invasion and induce apoptosis of ovarian cancer cells. This investigation may shed light on the immunotherapy potential of UCB-derived neutrophils on ovarian cancer cells. However, the molecular mechanism of UCB-derived neutrophils inhibits the progression of ovarian cancer cells that were not detected in this study, and we need to do further *in vivo* investigation to verify this finding and *in vitro* experiment for clinical application of adoptive cell therapy.

## Abbreviations

UCB: umbilical cord blood
OC: ovarian cancer
PBS: phosphate-buffered saline
NC: blank control group
GAPDH: glyceraldehyde-3-phosphate dehydrogenase
qRT-PCR: quantitative real-time PCR
BAX: BCL2-associated X
BCL2: B-cell lymphoma 2 protein
MMP2: matrix metalloprotein2
